# Supplementary material for: ERAP2 Increases the Abundance of a Peptide Submotif Highly Selective for the Birdshot Uveitis-Associated HLA-A29
Source: Front Immunol. 2021 Feb 25;12:634441. doi: 10.3389/fimmu.2021.634441 (PMC7950316; doi:10.3389/fimmu.2021.634441)
Supplement: Supplementary file 1 [file Image_1.pdf]

Supplemental Figures for:

ERAP2 increases the abundance of a peptide submotif highly selective for the Birdshot Uveitis-associated HLA-A29

W.J. Venema[1,2], S. Hiddingh[1,2], J.H. de Boer[1], F.H.J. Claas[3], A Mulder[3], A.I. Den Hollander[4], E. Stratikos[5], S. Sarkizova[6,7], L.T. van der Veken[8], G.M.C. Janssen[9], P.A. van Veelen[9], J.J.W. Kuiper[1,2*]

1. Department of Ophthalmology, University Medical Center Utrecht, University of Utrecht, Utrecht, The Netherlands.
2. Center for Translational Immunology, University Medical Center Utrecht, University of Utrecht, Utrecht, The Netherlands.
3. Department of Immunology, Leiden University Medical Center, Leiden, The Netherlands
4. Department of Ophthalmology, Donders Institute for Brain, Cognition and Behaviour, Department of Human Genetics, Radboud University Medical Center, Nijmegen, The Netherlands.
5. Department of Chemistry, National and Kapodistrian University of Athens, Panepistimiopolis Zographou 157 84, Greece.
6. Department of Biomedical Informatics, Harvard Medical School, Boston, MA, USA.
7. Broad Institute of MIT and Harvard, Cambridge, MA, USA.
8. Department of Genetics, Division Laboratories, Pharmacy and Biomedical Genetics, University Medical Center Utrecht, University of Utrecht, Utrecht, The Netherlands
9. Center for Proteomics and Metabolomics, Leiden University Medical Center, Leiden, The Netherlands.

* Corresponding author; email: J.J.W.Kuiper@umcutrecht.nl

Supplemental Figures

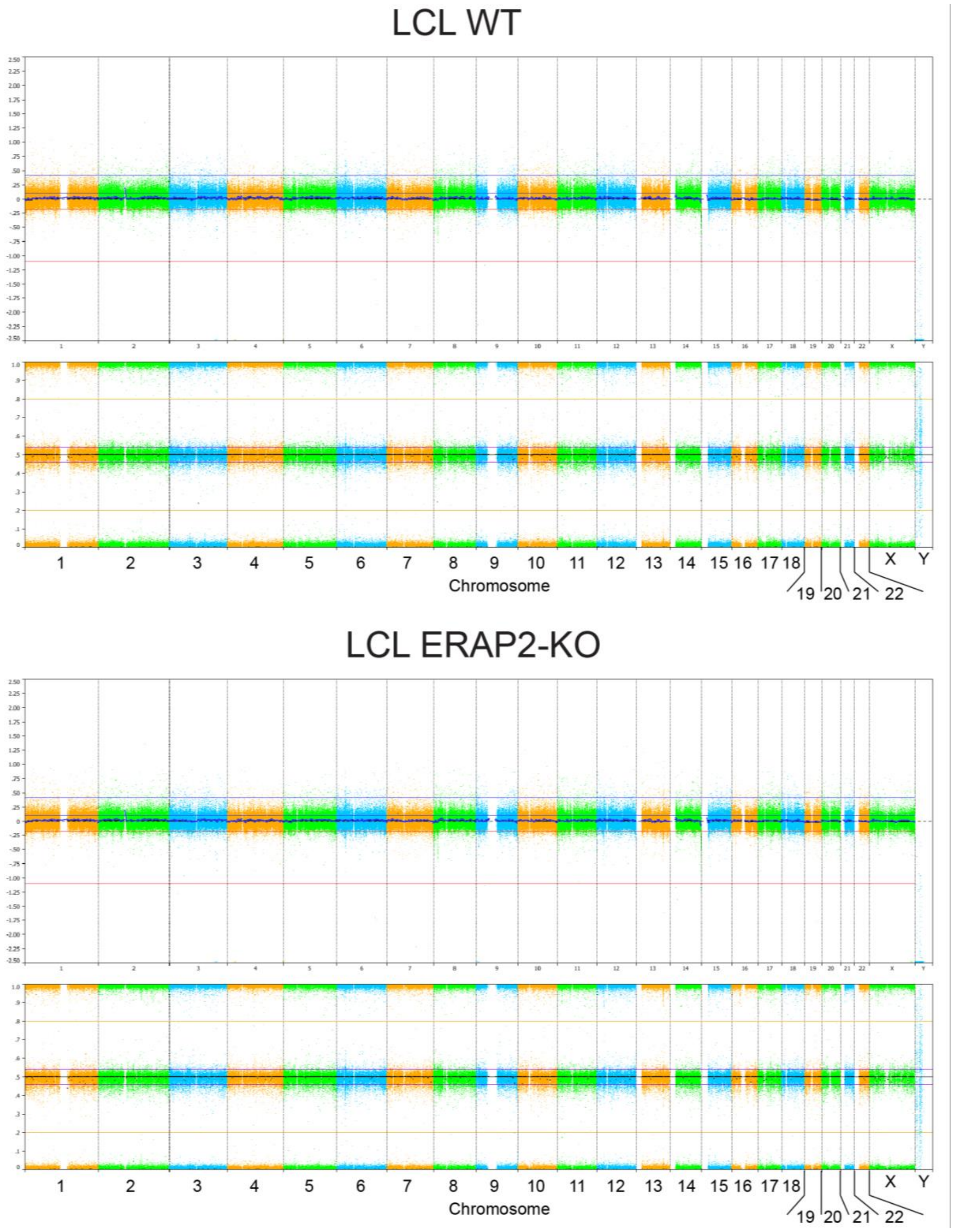

Supplemental Figure S1. Whole genome analysis using SNP arrays on unedited (LCL Wildtype, WT) and edited (ERAP2-KO) cell lines used in this study. SNPs were detected by the Infinium Human CytoSNP-850K v1.1 BeadChip (Illumina, San Diego, CA, USA) and show highly consistent genomes. The panels show the array results for the whole. On the X-axis the chromosomes and chromosomal region are indicated. The upper Y-axis shows the Log2 R ratio and the lower Y-axis indicates the B allele frequency for each SNP.

LCL WT

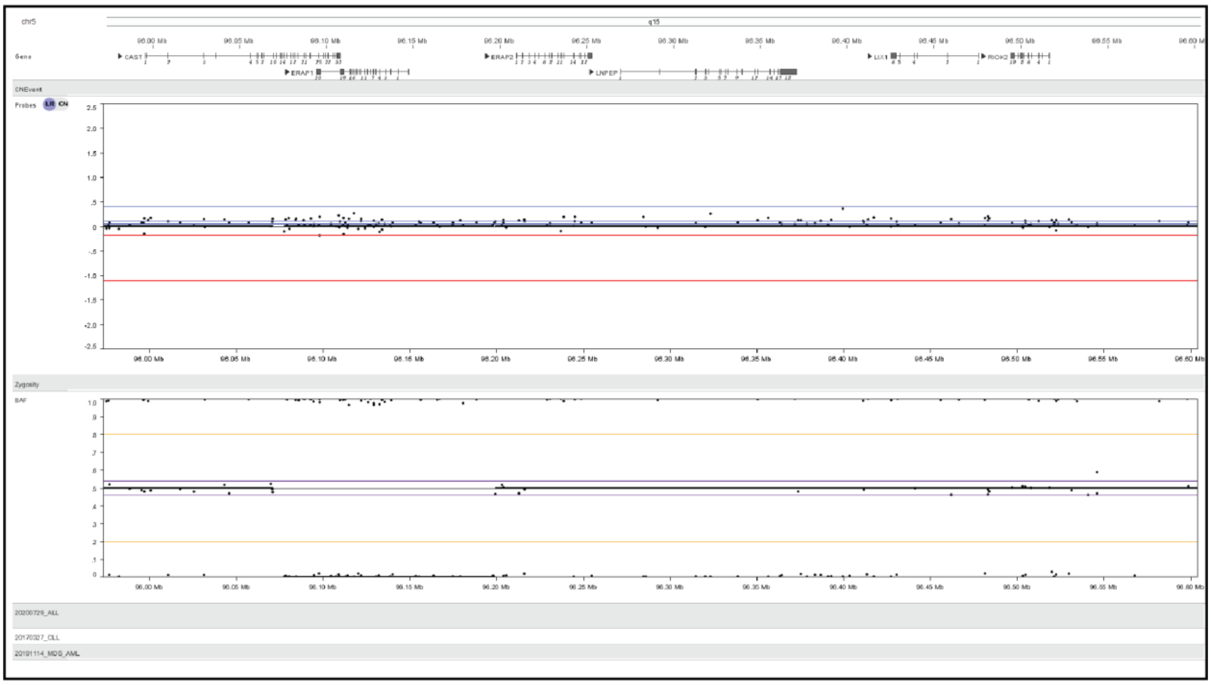

LCL ERAP2-KO

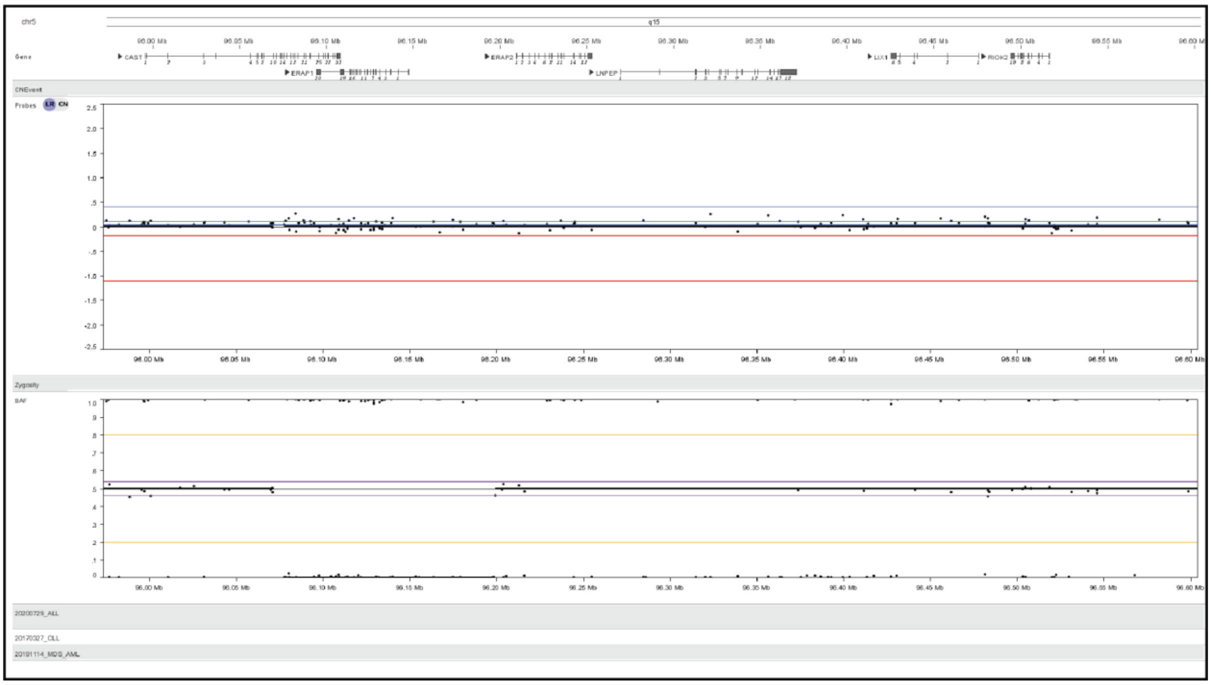

Supplemental Figure S2. Whole genome homozygosity analysis of unedited (LCL Wildtype, WT) and edited (ERAP2-KO) cell line using the Infinium Human CytoSNP-850K v1.1 BeadChip (Illumina, San Diego, CA, USA), similar to **Supplemental Figure S1**, but here the panels show the array results for the region near *5q15* including *ERAP1*, *ERAP2*, and *LNPEP*. The SNP probes are indicated by black dots. The upper Y-axis shows the Log2 R ratio for the probes and the lower Y-axis indicates the B allele frequency for each SNP (BAF).

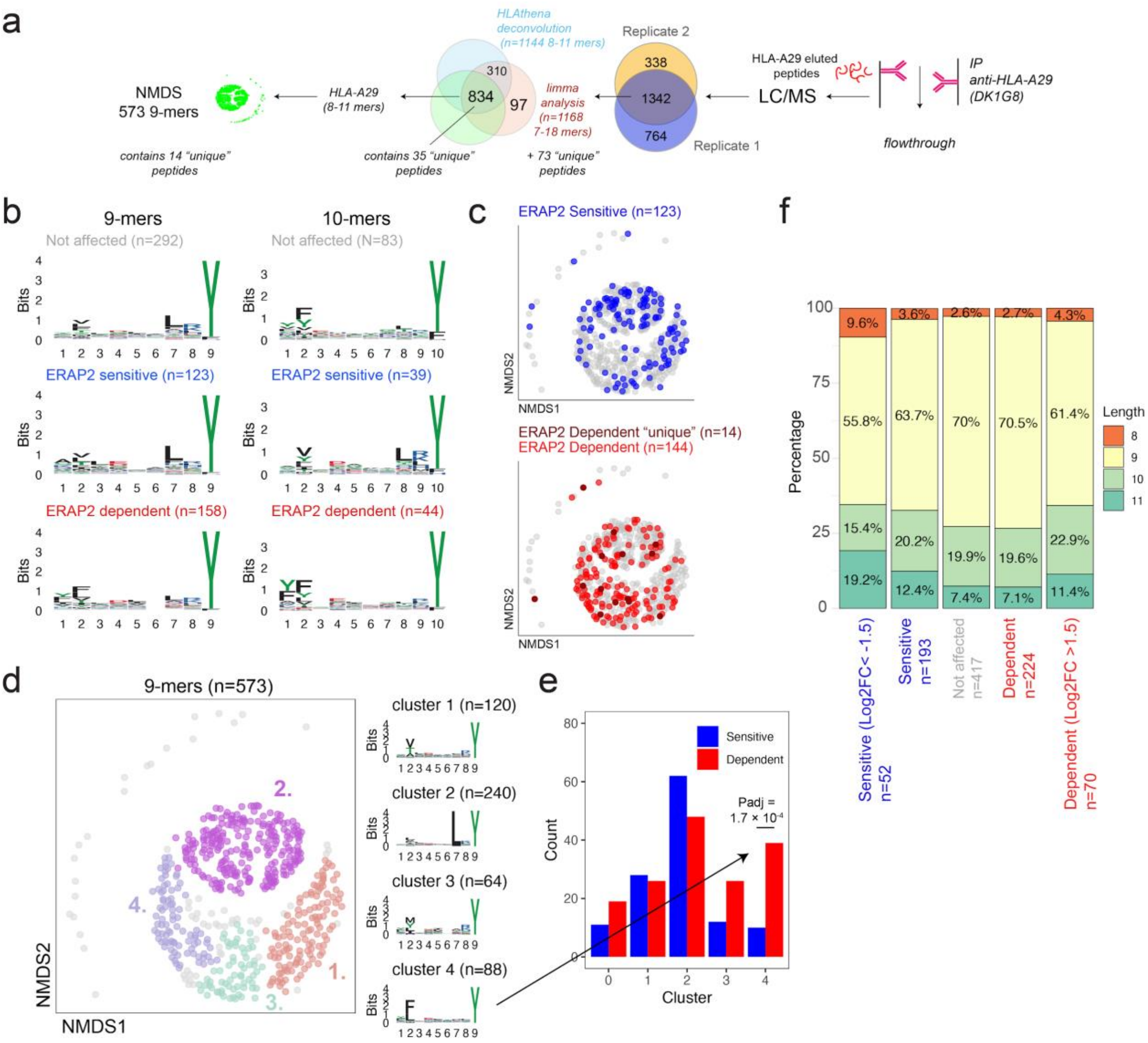

Supplemental Figure S3. HLA-A29 peptidome data analysis including peptides unique to either ERAP2-KO or ERAP2-WT cells. **a**) A total of 1342 peptides overlapping between the two biological replicates with percolator q<0.01 and Mascot Ions score >30, were filtered according to the steps indicated. Note that after the limma analysis, the 73 "unique" peptides detected in either the heavy or light labeled conditions (with consistent detection in the same channel in both experiments) were added to the dataset before deconvolution with *HLAthena* to filter for HLA-A29 ligands. **b**) The sequence logos for 9-mers and 10-mers in this dataset. ERAP2-sensitive peptides are peptides that decrease in amount in the presence of ERAP2 and ERAP2-dependent peptides increase in amount in the presence of ERAP2. **c**) Nonmetric multidimensional scaling of 573 9-mers in this dataset. The ERAP2-sensitive and ERAP2-dependent peptides are indicated in blue and red, respectively. Peptides uniquely identified in the ERAP2 WT-condition are shown in dark red (n=14). **d**) Four clusters were estimated (eps parameter for DBSCAN, using k=5) using the elbow method. The sequence logos for each cluster are indicated on the right. **e**) Comparison of the number of ERAP2-sensitive and ERAP2-dependent peptides in each peptide cluster identified in *b*. *Padj* = bonferroni corrected (n=clusters) *P* values from X^2 tests. All other comparisons were Padj>0.05. **f**) The percentage of 8-11-mers in peptides sets of this dataset. This analysis shows length dependent effects seen for ERAP2 in an hypoactive ERAP1 background.

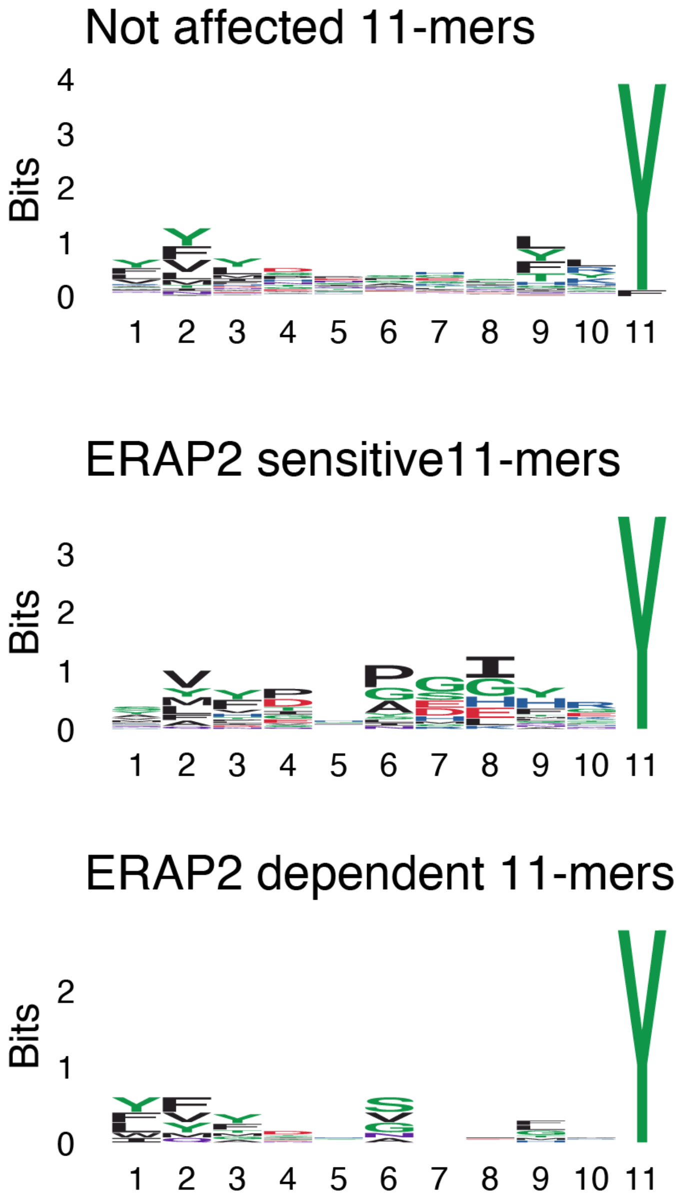

Supplemental Figure S4. The sequence logos for non-redundant 11-mers from HLA-A29. Peptides that decrease in the presence of ERAP2 are termed ERAP2-sensitive, peptides that increased in relative amounts are termed ERAP2-dependent. Peptides that did not change in relative amounts in the presence of ERAP2 are termed 'not affected'.

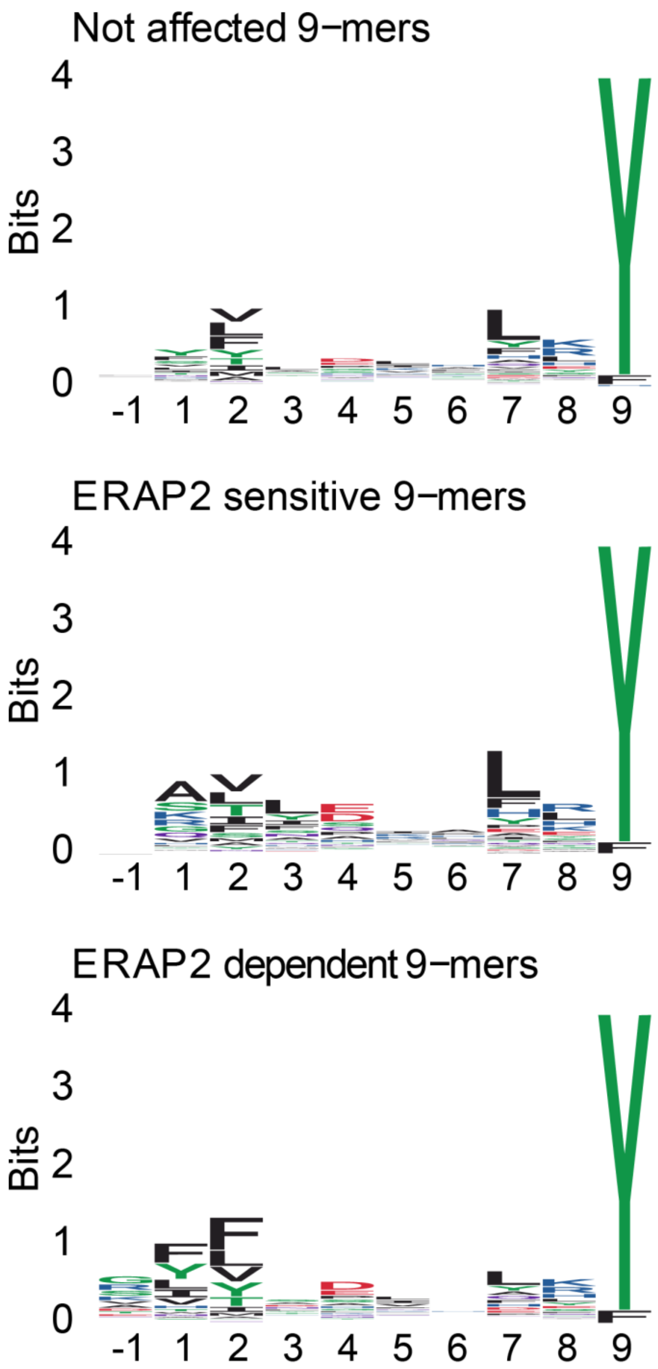

Supplemental Figure S5. The sequence logos for 948 non-redundant 9-mers and their designated P-1 derived from the amino acid sequence of the putative proteins. Peptides that decrease in the presence of ERAP2 are termed ERAP2-sensitive, peptides that increase in abundance are termed ERAP2-dependent. Peptides that did not change in abundance in the presence of ERAP2 are termed ‘not affected’.

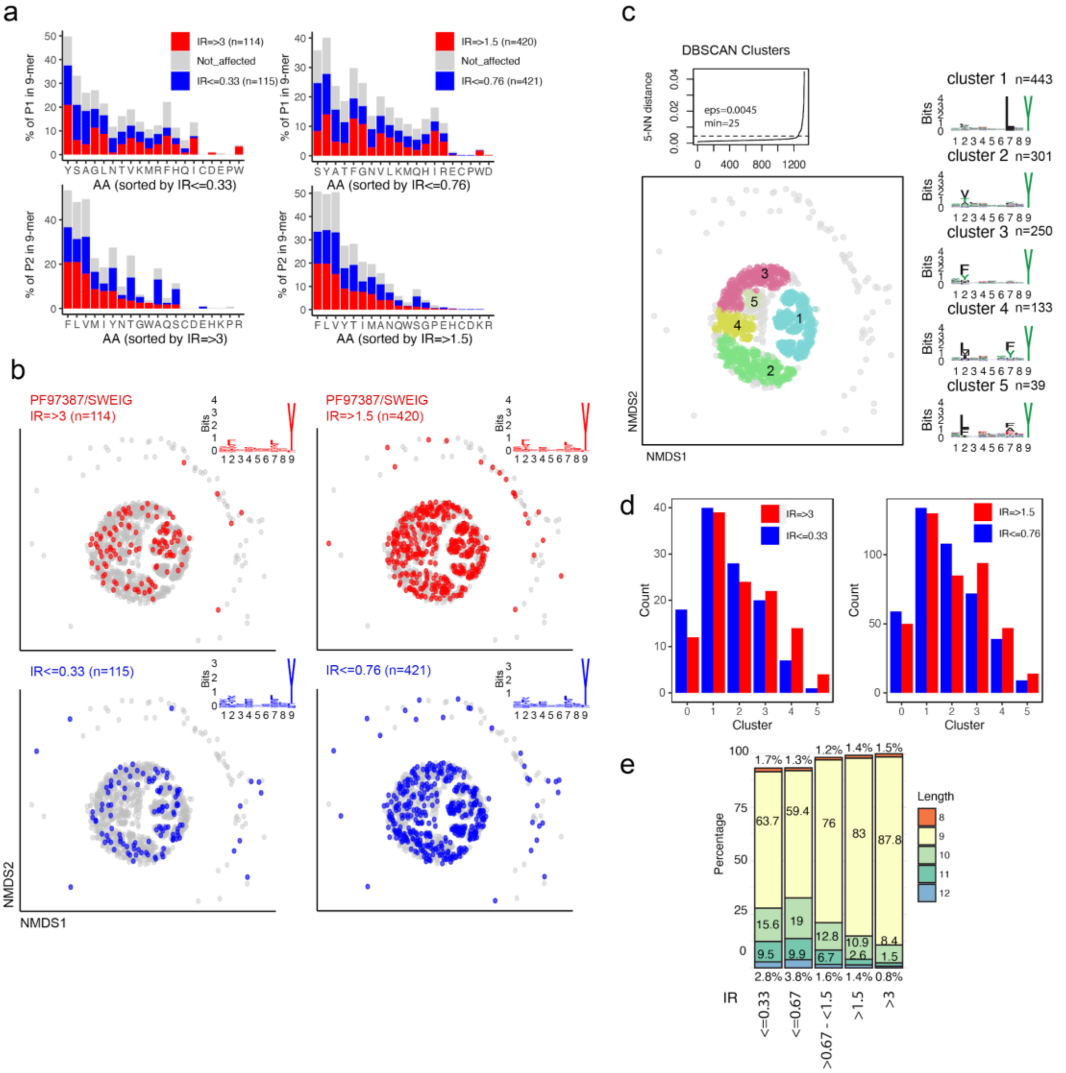

Supplemental Figure S6. **Non-metric multidimensional scaling of the 1329 shared 9-mers eluted from the HLA-A29-positive cell lines PF97387 (ERAP1 high expression/activity) and SWEIG (ERAP1 low expression/activity)**. The 1329 9-mers were filtered (removed peptides with value 0 in any of the 3 replicates from PF97387 or SWEIG) from a total of 5584 (3828 9-mers) peptides from *Alvarez-Navarro et al.,2015*. In this study, the normalized intensity ratio (PF97387/SWEIG) of each peptide in the two cell lines was used to infer the relative abundance of each peptide, which we adapted to assign peptides as ERAP2-dependent (IR ≥ 1.5 or IR ≥ 3) or ERAP2-sensitive (IR ≤ 0.76 or IR ≤ 0.33). We used IR≤ 0.76 (instead of 0.67) compared to IR ≥ 1.5 so the peptide datasets would be of equal size. **a)** Comparison of amino acid proportion at P1 and P2 of 9-mers (in percentage for each group of peptides) between peptides that decrease in abundance (in blue) in the presence of ERAP1 or that increase in abundance (red), compared to peptides not affected by ERAP1 cells (in grey). All comparisons were not significant; *Padj*>0.05. **b)** Non-metric multidimensional scaling of the 1329 9-mers **c)** Five clusters were estimated (eps parameter for DBSCAN, using k=5, based on Figure 3C) using the elbow method. The sequence logos for each cluster are indicated on the right. **d**) Comparison of the number of ERAP1-dependent (IR ≥ 1.5 or IR ≥ 3) and ERAP1-sensitive peptides (IR ≤ 0.76 or IR ≤ 0.33) in each peptide cluster identified in *b. Padj* = bonferroni corrected (n=clusters) X^2 tests. The difference between the count of sensitive and dependent peptides in each cluster was not significant or *Padj*>0.05. **e**) The percentage of 9-mers and 10-mers in peptides sets using different cut-offs for the intensity ratio (IR). This analysis confirms the length effects seen for ERAP1 as reported by *Alvarez-Navarro et al., 2015* in these cell lines (which are ERAP2-deficient).

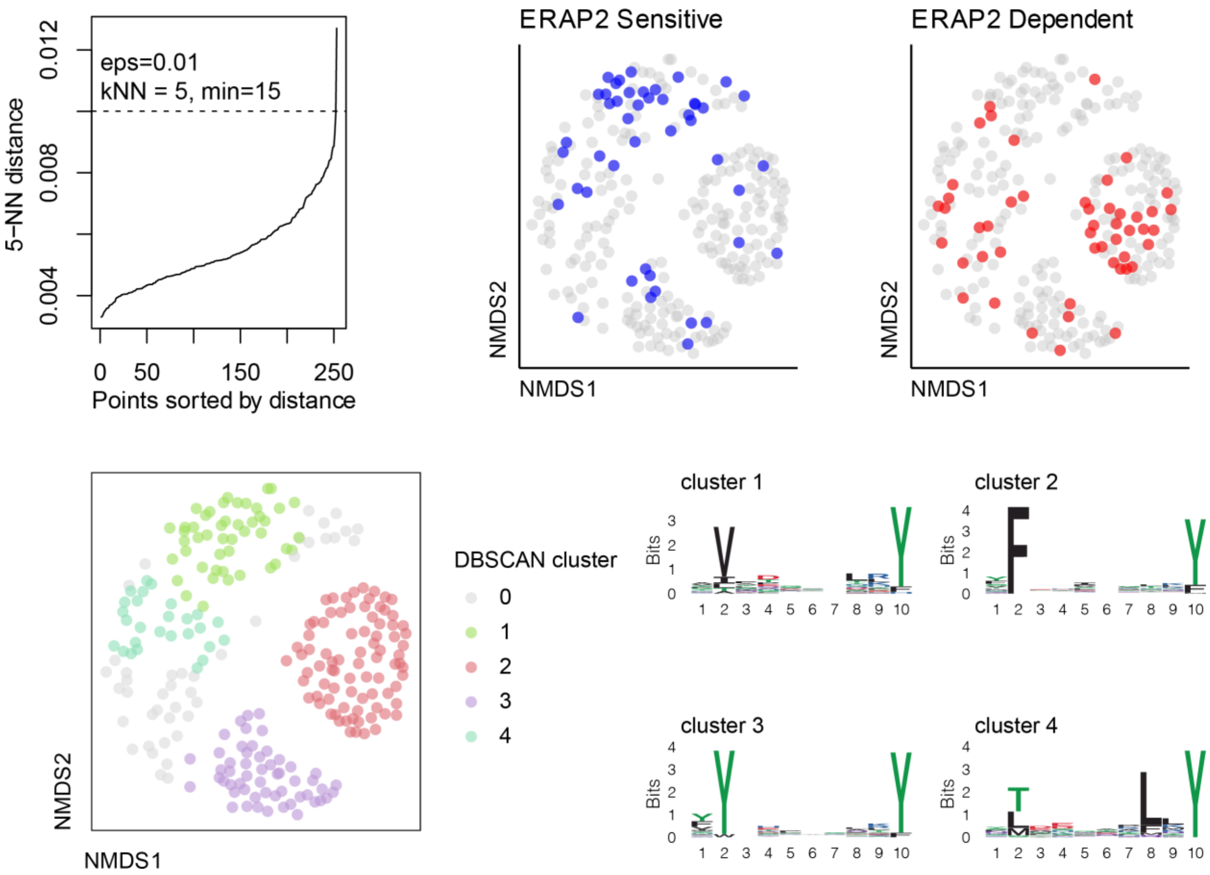

Supplemental Figure S7. Non-metric multidimensional scaling plot of 235 10-mers eluted from HLA-A29:02. Differentially expressed peptides are indicated in blue (ERAP2 sensitive that decrease in abundance in the presence of ERAP2) and red (ERAP2-dependent peptides that increase in abundance in the presence of ERAP2). A total of four clusters were identified and the sequence logos for each cluster are indicated. Cluster 0 indicates the unassigned peptides. 10-mer peptides of cluster 2 also show the P2-F motif for HLA-A29 and contains enrichment for ERAP2-dependent peptides compared to ERAP2-sensitive peptides, which reflects the results from 9-mers in **Figure 3**.

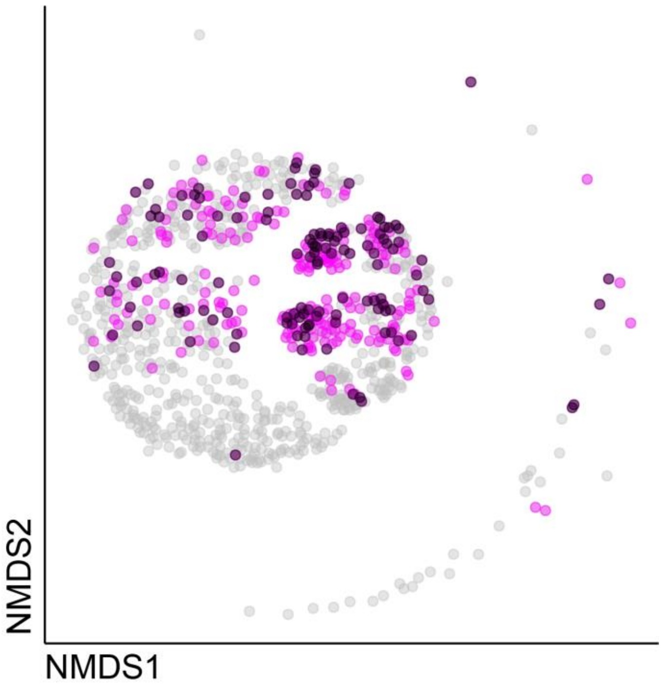

Supplemental Figure S8. Non-metric multidimensional scaling plot of 948 9-mers eluted from HLA-A29:02 in this study. Peptides with a binding score MSi>0.6 for HLA-A03:01 from *HLAthena* (https://HLAthena.tools) are highlighted in magenta. Peptides with a binding score MSi>0.6 for HLA-A03:01 that are differentially expressed (moderate q<0.01) are indicated in black.

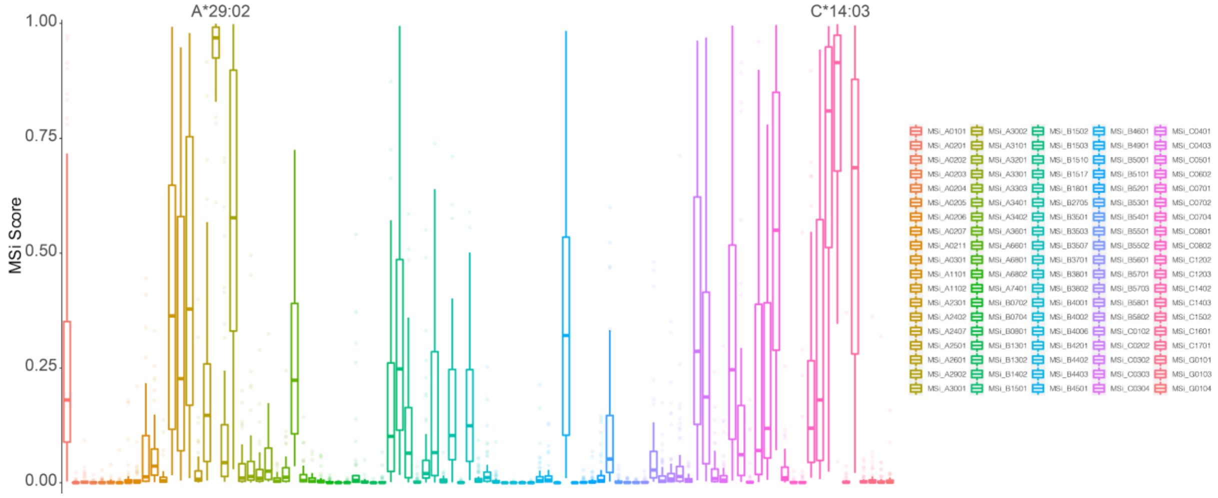

Supplemental Figure S9. Predicted binding scores (in MSi from HLAthena) for the 53 ERAP2-dependent peptides in cluster 2 (**Figure 3C**) across 95 HLA alleles (selection of alleles tested based on Sarkizova *et al.*, *2020*).

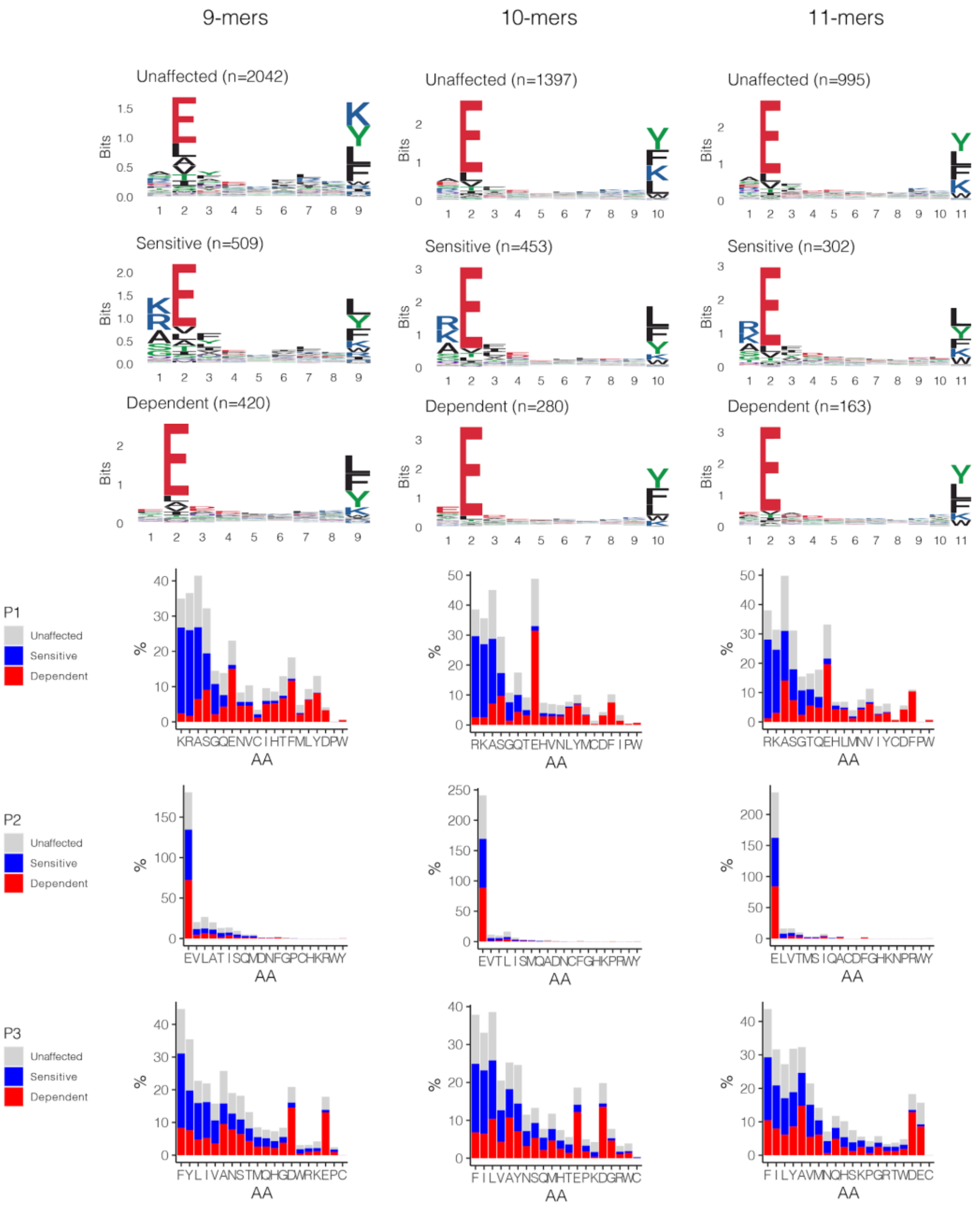

Supplemental Figure S10. The effect of ERAP2 on the HLA class I peptidome. Sequence motifs depict specific amino acid preferences for 9-, 10-, and 11-mers were generated from a non-redundant list of peptides from HLA class I (W6/32). Comparison of amino acid proportion at P1, P2, and P3 of (in percentage for each group of peptides) between peptides that decrease in abundance (‘sensitive’), peptides that increase in abundance (‘dependent’ peptides) compared to peptides not affected in ERAP2-WT cells (in grey).

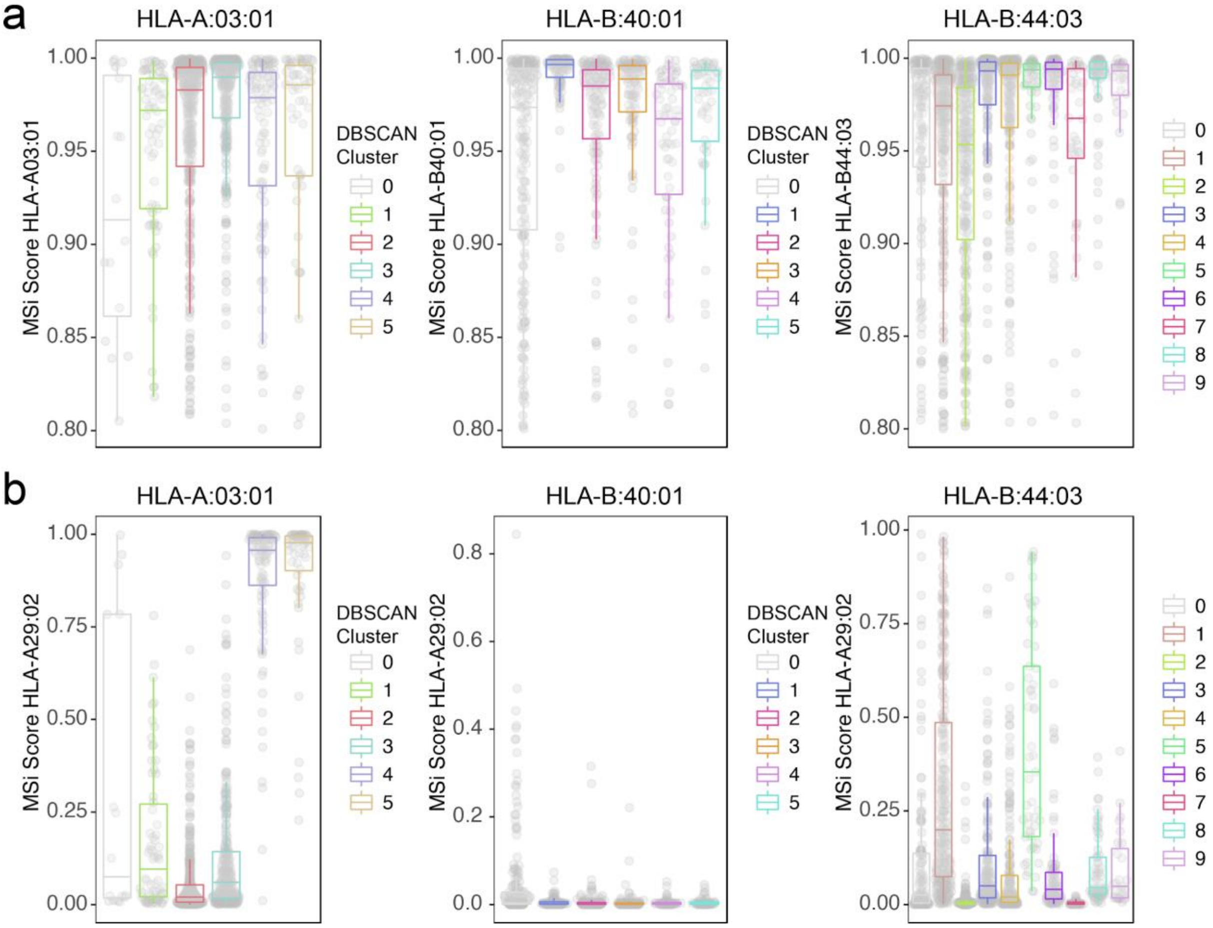

Supplemental Figure S11. Peptide bindings scores from *HLAthena* (HLAthena.tools) for peptide clusters from **Figure 4**. **a**) The binding score (MSi) for 9-mers with a MSi>0.8 used for the non-metric multidimensional scaling of *HLA-A*03:01*, *HLA-B*40:01*, and *HLA-B*44:03*. The binding score ranges from 0 (low) to 1 (high). Clusters identified by DBSCAN are indicated and color-coded. **b**) The binding score for *HLA-A*29:02* (MSi) for the same 9-mers and clusters as shown in *a*.

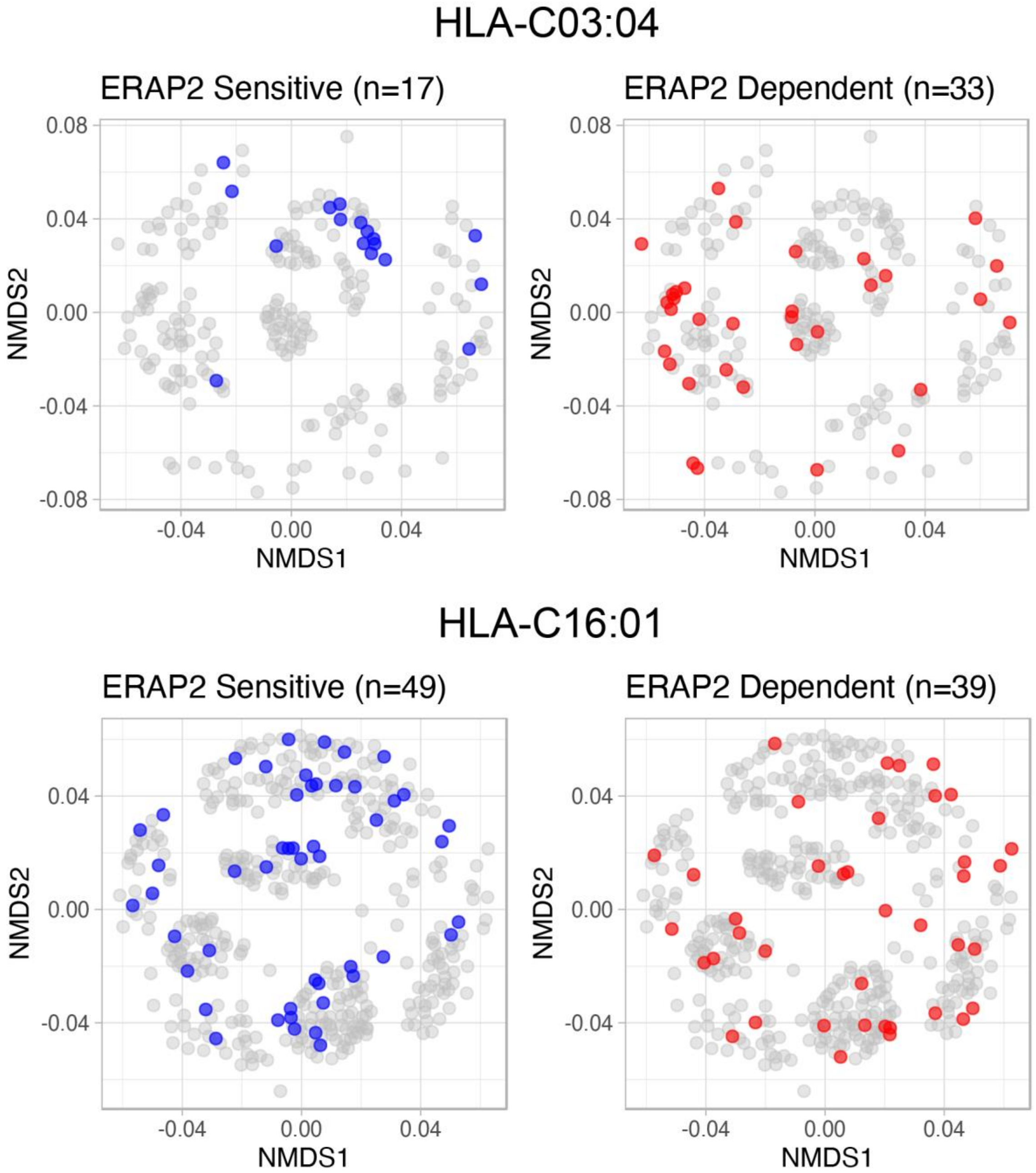

Supplemental Figure S12. Non-metric multidimensional scaling plot of 221 and 411 9-mers from HLA-C03:04 and HLA-C16:01. Differentially expressed peptides are indicated in blue (ERAP2 sensitive that decrease in abundance in the presence of ERAP2) and red (ERAP2-dependent peptides that increase in abundance in the presence of ERAP2).

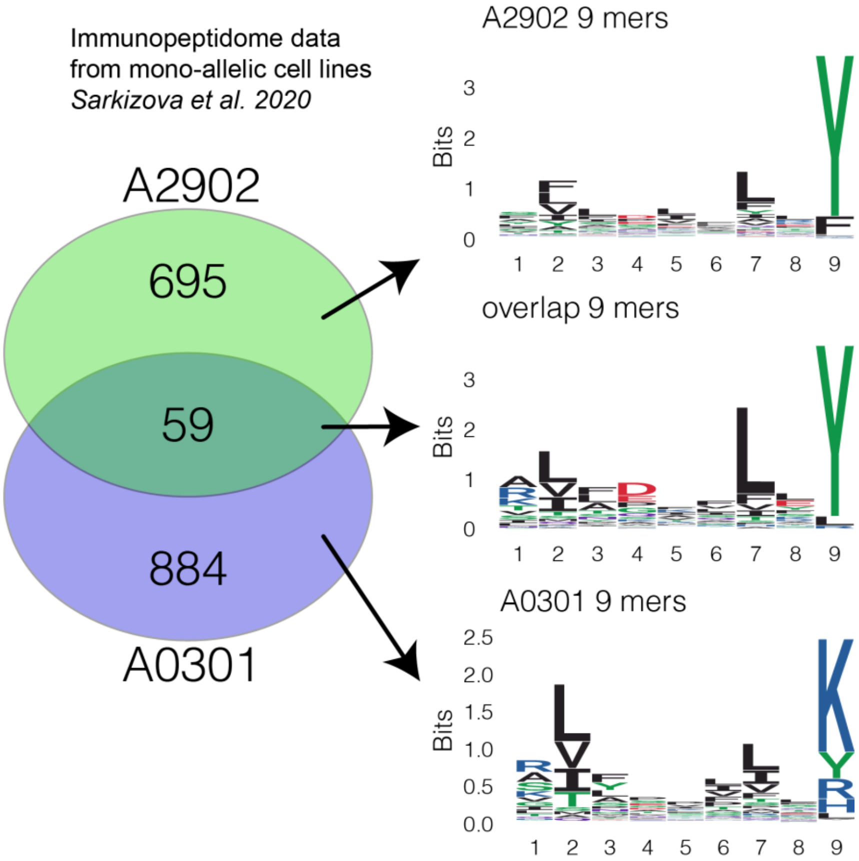

Supplemental Figure S13. Venn diagram of 9-mers presented by monoallelic cell lines expressing only HLA-A29:02 or only HLA-A03:01 from *Sarkizova et al., 2020* A total of 59 9-mers were detected in both datasets. The sequence logos for peptides uniquely observed in HLA-A29, overlapping peptides found in both monoallelic datasets, and peptides uniquely observed in HLA-A03 are indicated on the right.

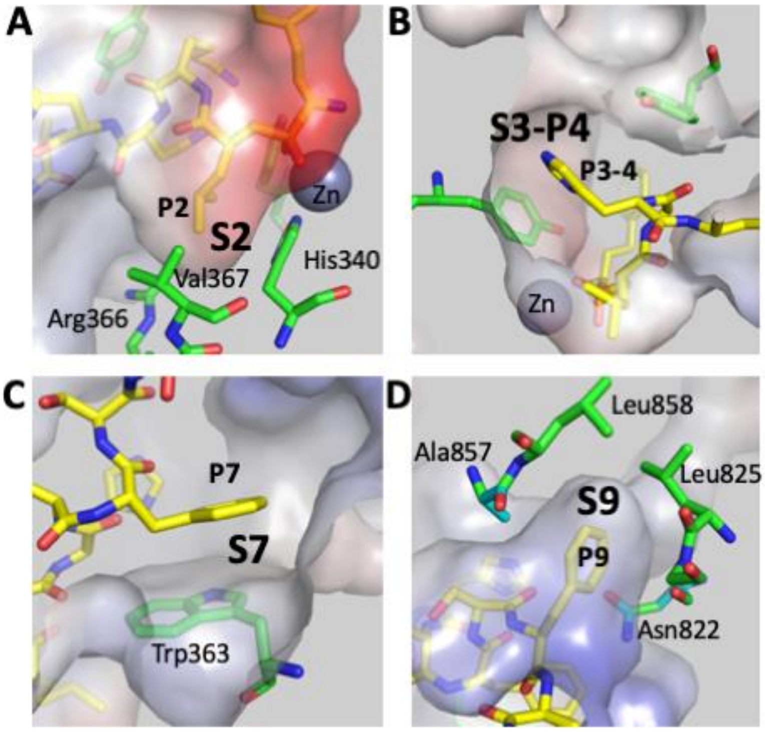

Supplemental Figure S14. Putative specificity pockets of ERAP2 that help explain observed sequence motifs. ERAP2 (from PDB code *5AB0*) is shown in surface representation colored by electrostatic potential (red=negative, white=neutral, blue=negative). Peptide analogue DG025 that was crystallized bound onto ERAP2 is shown in yellow sticks (carbon=yellow, oxygen=red, nitrogen=blue). Nearby ERAP2 residues that help form indicated specificity pockets are shown in green sticks. Specificity pockets are indicated as **a**) S2, **b**) S3-P4, **c**) S7 and **d**) S9. Peptide residue side-chains that are accommodated in the pockets are indicated as P2, P3-4, P7 and P9.

References

Alvarez-Navarro C, Martín-Esteban A, Barnea E, Admon A, López de Castro JA. Endoplasmic Reticulum Aminopeptidase 1 (ERAP1) Polymorphism Relevant to Inflammatory Disease Shapes the Peptidome of the Birdshot Chorioretinopathy- Associated HLA-A*29:02 Antigen. Mol Cell Proteomics. 2015 Jul;14(7):1770-80.

Brosch M, Yu L, Hubbard T, Choudhary J. Accurate and sensitive peptide identification with Mascot Percolator. J Proteome Res. 2009 Jun;8(6):3176-81.

Kammers K, Cole RN, Tiengwe C, Ruczinski I. Detecting Significant Changes in Protein Abundance. EuPA Open Proteom. 2015 Jun;7:11-19.

Sanz-Bravo A, Martín-Esteban A, Kuiper JJW, García-Peydró M, Barnea E, Admon A, López de Castro JA. Allele-specific Alterations in the Peptidome Underlie the Joint Association of HLA-A*29:02 and Endoplasmic Reticulum Aminopeptidase 2 (ERAP2) with Birdshot Chorioretinopathy. Mol Cell Proteomics. 2018 Aug;17(8):1564-1577.

Sarkizova S, Klaeger S, Le PM, Li LW, Oliveira G, Keshishian H, Hartigan CR, Zhang W, Braun DA, Ligon KL, Bachireddy P, Zervantonakis IK, Rosenbluth JM, Ouspenskaia T, Law T, Justesen S, Stevens J, Lane WJ, Eisenhaure T, Lan Zhang G, Clauser KR, Hacohen N, Carr SA, Wu CJ, Keskin DB. A large peptidome dataset improves HLA class I epitope prediction across most of the human population. Nat Biotechnol. 2020 Feb;38(2):199-209.
