# Supplementary material for: ERAP2 Increases the Abundance of a Peptide Submotif Highly Selective for the Birdshot Uveitis-Associated HLA-A29
Source: Front Immunol. 2021 Feb 25;12:634441. doi: 10.3389/fimmu.2021.634441 (PMC7950316; doi:10.3389/fimmu.2021.634441)
Supplement: Supplementary file 2 [file Image_2.pdf]

Supplemental Info for:

ERAP2 increases the abundance of a peptide submotif highly selective for the Birdshot Uveitis-associated HLA-A29

W.J. Venema[1,2], S. Hiddingh[1,2], J.H. de Boer[1], F.H.J. Claas[3], A Mulder[3], A.I. Den Hollander[4], E. Stratikos[5], S. Sarkizova[6,7], L.T. van der Veken[8], G.M.C. Janssen[9], P.A. van Veelen[9], J.J.W. Kuiper[1,2*]

1. Department of Ophthalmology, University Medical Center Utrecht, University of Utrecht, Utrecht, The Netherlands.
2. Center for Translational Immunology, University Medical Center Utrecht, University of Utrecht, Utrecht, The Netherlands.
3. Department of Immunology, Leiden University Medical Center, Leiden, The Netherlands
4. Department of Ophthalmology, Donders Institute for Brain, Cognition and Behaviour, Department of Human Genetics, Radboud University Medical Center, Nijmegen, The Netherlands.
5. Department of Chemistry, National and Kapodistrian University of Athens, Panepistimiopolis Zographou 157 84, Greece.
6. Department of Biomedical Informatics, Harvard Medical School, Boston, MA, USA.
7. Broad Institute of MIT and Harvard, Cambridge, MA, USA.
8. Department of Genetics, Division Laboratories, Pharmacy and Biomedical Genetics, University Medical Center Utrecht, University of Utrecht, Utrecht, The Netherlands
9. Center for Proteomics and Metabolomics, Leiden University Medical Center, Leiden, The Netherlands.

* Corresponding author; email: J.J.W.Kuiper@umcutrecht.nl

Supplemental Info:

Lentiviral vector production

HEK-293T cells were seeded into 10 cm dishes (2×10^6 cells/dish) and cultured in Dulbecco's Modified Eagle Medium (DMEM, Thermo Fisher Scientific). The next day, 293T cells were co-transfected with 2 µg transfer vector (Lenti ORF clone of Human S-antigen mGFP tagged, RC220057L2 from Origene) and components of 2nd generation packaging vectors: 8.33 µg psPAX2 packaging vector and 2.77 µg pMD2.G envelope vector at a ratio of 4:1. Transfection was done in serum-free DMEM using Lipofectamine 2000 (Thermo Fisher Scientific) according to manufacturer's instructions. Medium was replaced with 10 mL DMEM supplemented with 10% FBS and incubated at 37°C, 5% CO_2 after 24 hours. The conditioned medium containing lentiviral particles was collected 48 hours after transfection and an additional 10 mL of fresh culture medium was added to the cells. After 12 hours, harvested supernatants were combined and cleared by centrifugation at 1500 rpm for 5 minutes at 4°C then passed through a 0.45 µm filter. Lentiviral supernatants were concentrated using ultracentrifugation with a Beckman Coulter Optima centrifuge using a SW32Ti rotor. Filtered supernatant was added to 38.5 mL Ultra-Clear tubes (Beckman Coulter). Centrifugation was performed for 120 minutes at 32,000 rpm. Supernatant was completely removed and virus pellets were resuspended in 1 mL RPMI (containing 10% FBS and 1% penicillin/streptomycin) and stored at -80°C.

Lentiviral transduction of S-antigen in EBV-LCL

To obtain stable cell lines overexpressing S-antigen, EBV-LCLs were transduced with the concentrated lentiviral supernatants. To transduce EBV-LCLs, $1\text{x}10^6$ cells were seeded in a 24-wells plate with the lentivirus and a final polybrene concentration of 6 µg/mL. After 24 hours, the medium was replaced and the cells were cultured for another 3 days, without exceeding a cell concentration of 1.5×10^6 cells/mL. Transduction efficiency was monitored by fluorescent light microscopy. GFP-positive EBV-LCLs were sorted using the BD FACSAria™ III sorter and S-antigen expression levels were detected by western blot. Western blot analysis to detect the fusion protein S-antigen-GFP was done as described in the Method section. Figure 1 below shows the high protein expression of S-antigen and Tubulin detected from the same cell lysate samples of the patient-derived LCL, but run on two separate blots in parallel because the proteins are detected in close proximity on the blot.

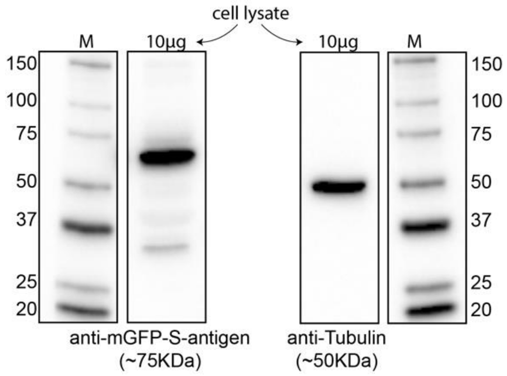

Figure 1. Westernblot analysis of retinal S-antigen after transduction of the patient-derived LCLs.

Differential expression analysis of peptides using limma

For differential expression analysis we used the workflow from Kammers *et al.*, 2015 available at http://www.biostat.jhsph.edu/~kkammers/software/eupa/R_guide.html. Their method exploits the R package *limma* for shrinking a peptide's sample variance towards a pooled estimate that boosts power for stable detection of (truly) significant changes in small proteomic data sets. Peptide data were preprocessed using the *read.peptides()* function, which excludes peptides with missing values (i.e., not detected in either the light or heavy channel). We computed dummy variables for the "I*solation.Interference*", "*Quan.Usage*", "*Quan.info*" variables, because quality control of the input data was completed as described in the main manuscript. The peptide sequence was used as the "*Protein.Group.Accessions*" variable. Overlapping peptide data from the biological replicates were independently normalized using the *quantify.proteins()* function. Following the workflow of Kammers et al., we used peptides (with a Mascot Percolator q<0.01 in all analyses) detected in both biological replicates (i.e., peptides unique to one of the conditions are left out for normalization and statistical analysis). For example, for peptides detected by DK1G8 (anti-HLA-A29) with a *HLAthena* binding score [MSi]>0.6 for HLA-A*29:02 a total of 1330 peptides were detected in both channels, while 41 peptides in either the light or heavy channel (with consistent detection in the same channel in both experiments) and were not considered for statistical analyses. We blocked for batch effect (two independent experiments) in *limma* by including them in the design matrix. HLA-A29 peptidome analysis considering also peptides detected in either the heavy or light channels is provided in **Figure S3**. Here, we used dummy variables for the moderate q-value (set to 1×10^{-6}) and $\log_2$FC ($\log_2$FC= -6.6 for peptides only detected in the ERAP2 KO-cell line and $\log_2$FC=6.6 for peptides detected only in the ERAP2 WT-cell line), because these parameters were only used to subset peptides unique to either of the conditions (using moderate q<0.01 as a threshold) together with the differentially expressed peptides detected in both channels. Also, although Mascot Percolator exploits a number of relevant peptide features and has been shown to be superior in accurate peptide identification compared to previous Mascot scoring based on one metric (Borsch et al.,2009), we also conducted this analysis of the HLA-A29 peptidome using the percolator q-value in conjunction with the Mascot ions score >30, which showed similar effects for ERAP2 at the submotif level as the analysis using the percolator q-value (see **Figure S3**).

References Supplemental Info

Alvarez-Navarro C, Martín-Esteban A, Barnea E, Admon A, López de Castro JA. Endoplasmic Reticulum Aminopeptidase 1 (ERAP1) Polymorphism Relevant to Inflammatory Disease Shapes the Peptidome of the Birdshot Chorioretinopathy- Associated HLA-A*29:02 Antigen. Mol Cell Proteomics. 2015 Jul;14(7):1770-80.

Brosch M, Yu L, Hubbard T, Choudhary J. Accurate and sensitive peptide identification with Mascot Percolator. J Proteome Res. 2009 Jun;8(6):3176-81.

Kammers K, Cole RN, Tiengwe C, Ruczinski I. Detecting Significant Changes in Protein Abundance. EuPA Open Proteom. 2015 Jun;7:11-19.

Sanz-Bravo A, Martín-Esteban A, Kuiper JJW, García-Peydró M, Barnea E, Admon A, López de Castro JA. Allele-specific Alterations in the Peptidome Underlie the Joint Association of HLA-A*29:02 and Endoplasmic Reticulum Aminopeptidase 2 (ERAP2) with Birdshot Chorioretinopathy. Mol Cell Proteomics. 2018 Aug;17(8):1564-1577.

Sarkizova S, Klaeger S, Le PM, Li LW, Oliveira G, Keshishian H, Hartigan CR, Zhang W, Braun DA, Ligon KL, Bachireddy P, Zervantonakis IK, Rosenbluth JM, Ouspenskaia T, Law T, Justesen S, Stevens J, Lane WJ, Eisenhaure T, Lan Zhang G, Clauser KR, Hacohen N, Carr SA, Wu CJ, Keskin DB. A large peptidome dataset improves HLA class I epitope prediction across most of the human population. Nat Biotechnol. 2020 Feb;38(2):199-209.
